# Mechanisms of ADC Toxicity and Strategies to Increase ADC Tolerability

**DOI:** 10.3390/cancers15030713

**Published:** 2023-01-24

**Authors:** Toan D. Nguyen, Brandon M. Bordeau, Joseph P. Balthasar

**Affiliations:** Department of Pharmaceutical Sciences, University at Buffalo, Buffalo, NY 14214, USA

**Keywords:** antibody-drug conjugate, cancer, ADC toxicity and tolerability, targeted therapy

## Abstract

**Simple Summary:**

Antibody-drug conjugates (ADC) are a rapidly expanding class of anti-cancer drugs, with twelve agents in current clinical use. Despite recent successes, many ADCs fail during clinical development due to excessive toxicities and unfavorable risk-benefit profiles. Even for those ADCs that have been approved for clinical use, a substantial fraction of treated patients require dose reduction, treatment delays, or treatment discontinuation due to intolerable ADC-associated toxicity. In this report, we review the mechanisms contributing to the clinical toxicity of ADCs, and we discuss strategies to improve ADC tolerability.

**Abstract:**

Anti-cancer antibody-drug conjugates (ADCs) aim to expand the therapeutic index of traditional chemotherapy by employing the targeting specificity of monoclonal antibodies (mAbs) to increase the efficiency of the delivery of potent cytotoxic agents to malignant cells. In the past three years, the number of ADCs approved by the Food and Drug Administration (FDA) has tripled. Although several ADCs have demonstrated sufficient efficacy and safety to warrant FDA approval, the clinical use of all ADCs leads to substantial toxicity in treated patients, and many ADCs have failed during clinical development due to their unacceptable toxicity profiles. Analysis of the clinical data has demonstrated that dose-limiting toxicities (DLTs) are often shared by different ADCs that deliver the same cytotoxic payload, independent of the antigen that is targeted and/or the type of cancer that is treated. DLTs are commonly associated with cells and tissues that do not express the targeted antigen (i.e., off-target toxicity), and often limit ADC dosage to levels below those required for optimal anti-cancer effects. In this manuscript, we review the fundamental mechanisms contributing to ADC toxicity, we summarize common ADC treatment-related adverse events, and we discuss several approaches to mitigating ADC toxicity.

## 1. Introduction

Antibody-drug conjugates (ADCs) are a rapidly growing class of anti-cancer therapeutics, with more than 100 ADCs undergoing clinical investigation [1]. Currently, 12 ADCs have been approved by the United States Food and Drug Administration (FDA), including gemtuzumab ozogamicin (Mylotarg), brentuximab vedotin (Adcetris), inotuzumab ozogamicin (Besponsa), trastuzumab emtansine (Kadcyla), polatuzumab vedotin (Polivy), enfortumab vedotin (Padcev), trastuzumab deruxtecan (Enhertu), sacituzumab govitecan (Trodelvy), belantamab mafodotin (Blenrep), loncastuximab tesirine (Zynlonta), tisotumab vedotin (Tivdak), and mirvetuximab soravtansine (Elahere).

ADCs are composed of a monoclonal antibody (mAb) tethered to a cytotoxic small-molecule drug (i.e., “payload”) through a chemical linker. Most of the ADCs that have been investigated have employed payload molecules that have shown poor efficacy and substantial toxicity when administered as unconjugated (i.e., “free”) agents [2,3]. As anticipated by the pharmacokinetics and biodistribution of monoclonal antibody drugs [4,5], where high-affinity mAb binding to cell membrane proteins enables the localization of a substantial fraction of mAb to the targeted cell populations, the chemical conjugation of the payload to the anti-cancer mAb increases the selectivity of the delivery of the payload to the cancer cells, and thereby increases the therapeutic index of the payload [6]. However, despite recent successes, the clinical development of ADCs has been associated with a high failure rate, as off-site toxicity remains problematic, limiting tolerable ADC doses to levels below those required for substantial anti-cancer efficacy [7,8,9,10]. Even for the ADCs that have gained FDA approval, a significant fraction of treated patients require supportive treatment to reduce the severity of ADC-associated toxicities, and many patients require dose reduction, treatment delays, or treatment discontinuation [11]. 

The investigation of the safety profile of an ADC under development requires a series of preclinical and clinical studies; however, prior ADC development efforts suggest that the clinical toxicity profile of ADCs primarily relates to the payload component [12]. Since a relatively small group of payload molecules (i.e., MMAE, MMAF, DM1, DM4, calicheamicin, SN38, Dxd, PBD) are employed within the vast majority of approved ADCs and ADCs under development [8,13,14,15], consideration of the mechanisms associated with the known toxicities of previously developed ADCs may inform the development of new ADCs. In this manuscript, we provide an overview of the main mechanisms underlying ADC toxicity, and we provide a summary of the clinical safety profile of approved ADCs. Additionally, the manuscript discusses approaches to mitigating or preventing ADC toxicities.

## 2. Mechanisms of ADC Toxicity

**Figure 1:**
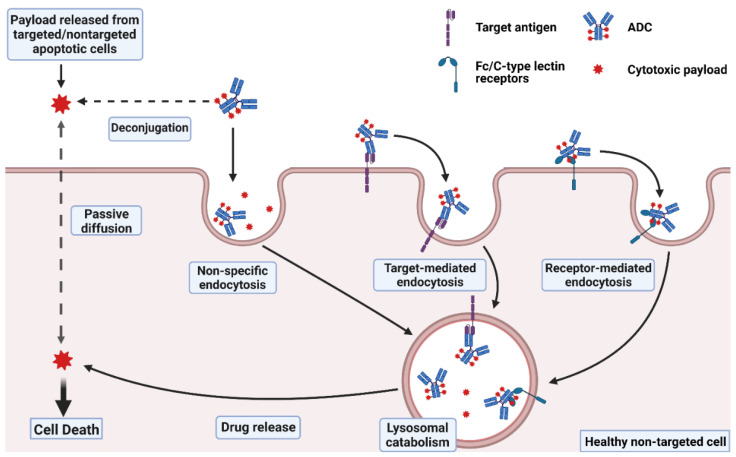
Mechanisms of ADC toxicity. Uptake of intact ADCs into normal cells may occur through non-specific endocytosis, or through internalization upon binding to the target antigen or to Fc/C-type lectin receptors. Payloads released from ADC deconjugation or other targeted/non-targeted apoptotic cells in the extracellular fluid may also enter normal cells via passive diffusion for membrane-permeable payloads or via non-specific endocytosis for membrane-impermeable linker-payload adducts. Created with BioRender.com.

It is approximated that only ~0.1% of the injected dose of an ADC is delivered to the targeted diseased cell population, with the vast majority of the administered dose catabolized “off-site” within non-targeted healthy cells, potentially leading to unwanted toxicities [16,17]. Off-site ADC toxicity may be categorized as “on-target” or “off-target”, where on-target toxicity proceeds through ADC binding to the targeted cell surface protein on healthy cells. Each component of the ADC, including the antibody, linker, and payload, may affect the extent of the ADC-induced toxicities. In this section, several mechanisms that lead to the toxicities of ADCs are discussed (Figure 1).

### 2.1. Target-Independent Toxicity: Off-Target, Off-Site Toxicity

Conceptually, ADCs are expected to enhance the selectivity of chemotherapy by facilitating the targeted delivery of cytotoxic payload molecules to the desired cell populations (on-target, on-site toxicity) while decreasing the delivery of the payloads to non-targeted healthy tissues, thus widening the therapeutic index. The anticipated safety concern for anti-cancer ADCs during the early phases of technology development was on-target (i.e., target-mediated) toxicity within tissues with some degree of expression of the target antigen, and the differential expression of the target in cancer cells versus in healthy tissues was expected to be the critical determinant of the therapeutic index of the ADCs [18]. However, clinical experience with ADCs subsequently demonstrated that the dose-limiting toxicities (DLTs) are rarely driven by target expression in healthy tissues. In a detailed review of preclinical and clinical data from 20 investigational new drug (IND) applications for ADCs filed between 2012 and 2013, the authors found that ADCs with the same class of linker/payload typically shared highly similar toxicity profiles, DLTs, and maximum tolerated doses (MTDs), regardless of the antigen targeted and regardless of the extent of the antigen’s expression in healthy tissues [15]. For example, the review discussed eight different ADCs under clinical development that incorporated the same vc-MMAE linker-payload composition. Each MMAE ADC was advanced to Phase II clinical investigation at a very similar dose (i.e., within the narrow range of 1.8 mg/kg to 2.4 mg/kg). All eight MMAE ADCs demonstrated similar DLTs (severe bone marrow toxicity, sepsis, and severe motor neuropathy). This same toxicity profile is shown for all FDA-approved vc-MMAE ADCs, including polatuzumab vedotin [19], enfortumab vedotin [20], and tisotumab vedotin [21]. Similarly, in a review of clinical ADC data published between 2010 and 2014, Masters et al. found that the prevalent grade 3/4 toxicities associated ADCs were consistent with their payload class [12]. For instance, severe anemia, neutropenia, and peripheral neuropathy were commonly reported for MMAE ADCs. For DM1 ADCs, grade 3/4 thrombocytopenia and hepatic toxicity are typically observed, and severe ocular toxicity is consistently reported for MMAF and DM4 ADCs. Recently, Saber and Leighton performed a follow-up analysis of 15 IND applications for ADCs containing PBD-dimer payloads submitted between 2013 and 2017. They found that the toxicity profiles of the PBD-ADCs were highly comparable, with common adverse events including vascular leak syndrome, elevated liver enzymes, bone marrow suppression, gastrointestinal events, metabolic effects, musculoskeletal events, neuropathy, pain, dyspnea, fatigue, and kidney injury [22]. The observations found in these studies were consistent with the findings from a recent systemic review and meta-analysis conducted by Zhu et. al. on common and severe treatment-related adverse events associated with ADCs in clinical trials between 2000 and 2022 [23]. These findings suggest that, to date, most of the off-site ADC toxicity relates to off-target delivery of the cytotoxic payload, and that off-target payload delivery is the critical driver for the tolerability of ADCs and, ultimately, the recommended doses used in patients. Of course, it is not completely unexpected that on-target toxicity is infrequently a major concern during the clinical evaluation and use of ADCs. Agents leading to severe on-target toxicity may be likely to be identified quickly during the early phases of preclinical development, leading to deselection prior to their advancement to clinical studies.

#### 2.1.1. Off-Target Delivery of ADC Payloads

Following ADC dosing, the released (i.e., “free”) payload rapidly appears within the systemic circulation. Plasma exposure to free payload relates, in part, to premature deconjugation of the payload in the systemic circulation (e.g., due to inadequate linker stability) [24]. There are two main classes of linkers: cleavable and non-cleavable. Cleavable linkers contain chemical or enzymatic liable chemistries formulated to exploit specific conditions unique to the intracellular or tumor extracellular environments, with the goal of maintaining good stability in the systemic circulation and rapid cleavage at the target site [25,26]. In practice, cleavable linkers are often hydrolyzed in plasma at an appreciable rate, leading to the premature release of the payload in the extra-tumoral compartments. Lipophilic payloads exhibit high permeability through plasma membranes and, consequently, the released payload enters non-targeted cells efficiently (e.g., via membrane diffusion), potentially leading to unwanted cytotoxicity. For example, the hydrazone linker used in the first-generation, calicheamicin-based ADC, gemtuzumab ozogamicin, was designed to be cleaved in the acidic environment of cellular lysosomes (i.e., following cell entry via receptor-mediated endocytosis). However, this linker exhibits an appreciable rate of hydrolysis in plasma, leading to substantial payload release prior to the ADC’s engagement with the targeted antigen, and decreasing the fraction of intact ADC delivered to targeted cells [25,27]. 

It is important to note that ADCs with cleavable linkers may be considered to be prodrugs, and it may be expected that 100% of the administered drug (i.e., payload) is eventually liberated through linker hydrolysis. In most cases, it may be expected that the payload is eliminated from the body by a clearance (CL) process such as renal filtration, biliary excretion, or hepatic biotransformation, where the pathway of elimination and the efficiency of the free payload CL is not influenced by the site of the payload release (i.e., within plasma due to premature linker hydrolysis, or within targeted or non-targeted cells following endocytosis). The fundamental pharmacokinetic theory predicts that the cumulative exposure to the released payload in plasma (e.g., as measured by the cumulative area under the free payload plasma concentration v. time curve, AUC) is a simple function of the ADC or payload dose and the payload CL (i.e., AUC = dose/CL). As such, poor linker stability is unlikely to influence payload AUC in plasma. However, poor linker stability is expected to decrease the ratio of payload exposure in targeted sites relative to plasma, such that poor linker stability is expected to decrease the ratio of on-site to off-site ADC cytotoxicity (decreasing efficacy relative to toxicity). With the recent advancement of linker technology, several ADCs have been developed using cleavable linkers with substantially improved stability (i.e., relative to those employed in first-generation ADCs, such as gemtuzumab ozogamicin). Yet, they still face the challenge of non-selective payload deconjugation in circulation due to the susceptibility of the linkers to plasma proteases for peptide-based linkers, to plasma reactive thiols for disulfide-based and maleimide-based linkers, or to serum esterases for alkyl carbamate linkers [27,28,29,30]. 

In contrast to cleavable linkers, non-cleavable linkers are more stable in plasma but require the complete intracellular proteolytic catabolism of the ADCs to yield cytotoxic metabolites. These metabolites typically consist of the intact linker-payload attached to the conjugating amino acid residue from the antibodies, such as lysine-SMCC-DM1 for trastuzumab emtansine or cysteine-MC-MMAF for belantamab mafodotin [25]. These metabolites are often charged and exhibit low permeability through cell membranes [31]. Although ADCs employing non-cleavable linkers generally show a more favorable tolerability, potentially due to reduced off-target toxicity relating to free payload exposure, ADCs with cleavable linkers typically demonstrate a superior efficacy [32]. This enhanced efficacy is partially attributed to the bystander effect, which refers to the ability of the free payload to diffuse from intracellular sites of ADC catabolism and payload release to neighboring cells within the local tumor environment [33]. ADCs with lipophilic payloads and cleavable linkers, which make up more than 80% of the currently approved ADCs, are the preferred choice for the treatment of cancers associated with heterogeneous antigen expression or slow rates of antigen/ADC internalization [32]. Besides amplifying the anti-tumor potency, the bystander effect can also exacerbate the off-target toxicities of an ADC due to the increased distribution of lipophilic membrane-permeable payloads in normal tissues. For instance, in a study by Polson et al., several ADCs were constructed by conjugating antibodies against a panel of non-Hodgkin lymphoma antigens to a DM1 payload via either a cleavable (SPP) or a non-cleavable (SMCC) linker and were tested for in vivo toxicity and efficacy [34]. The SMCC-ADCs showed efficacy against only two of the seven target antigens, while the SPP-ADCs were active against all targets. However, at the dose of 20 mg ADC/kg, the animals treated with an ADC bearing the cleavable linker SPP exhibited much more significant weight loss, hepatic toxicity, and hematological toxicities when compared with the results observed following dosing with ADC employing the non-cleavable linker SMCC. 

In addition to the entry of released payload into non-targeted cells via passive diffusion across plasma membranes, the non-specific endocytosis of the intact ADC may also contribute to the off-site delivery of payload. Non-specific endocytosis may be influenced by the physicochemical properties of ADCs, including hydrophobicity and charge. Since most of the drug-linker compositions utilized in ADC technology are highly lipophilic, the hydrophobicity of the ADCs is often proportional to the drug loading (i.e., drug-to-antibody ratio, DAR). In a study by Hamblett et al., several anti-CD30-vc-MMAE ADCs with DARs of two, four, and eight were evaluated for in vivo pharmacokinetics, efficacy, and toxicity [35]. It was observed that the ADCs with higher DAR values had a faster systemic clearance, a lower tolerability, and a narrower therapeutic index than the ADCs with a lower DAR. Similarly, Sun et al. demonstrated that maytansinoid-conjugated ADCs with a DAR of 10 had a 5-fold higher clearance and a decreased in vivo efficacy and tolerability compared to ADCs with a DAR lower than 6 [36]. Mice treated with low DAR ADCs (2 and 3.5) experienced less severe weight loss (approximately 4% nadir body weight loss) compared to ADCs with a DAR greater than 5.5 (7 to 9% nadir weight loss). In addition, they also observed a significantly elevated distribution of ADCs with higher DARs in the liver, possibly due to non-specific uptake by Kupffer cells and hepatic sinusoidal endothelial cells [32]. 

Positively charged molecules generally have increased charge-mediated endocytic uptake due to ionic attraction to negatively charged cell membranes [37]. Several preclinical studies have shown that the plasma clearance and tissue distribution of monoclonal immune gamma globulin (IgG) antibodies correlates with their isoelectric point (pI) [37]. For example, a study by Stuber et al. demonstrated that positively charged variants of an IgG1 antibody exhibited enhanced uptake in the liver and the spleen in mice and diminished plasma exposure compared to their neutral or negatively charged counterparts [38]. Another study by Liu et al. assessed the stability, cellular disposition, and in vivo disposition of trastuzumab (TS) mutants with incremental changes in pI from −14 to +17 [39]. They found that the positively charged variants (TS + 11, TS + 15, TS + 16, TS + 17) formed significant aggregates or failed to purify, and therefore they were excluded from subsequent experiments. In in vitro co-culture experiments with HER-2 and FcRn non-expressing Madin-Darby canine kidney cells, a significant increase in the intracellular accumulation of TS + 5 was observed compared to the negatively charged variants. Interestingly, their in vivo pharmacokinetic study indicated a U-shape relationship between charge and clearance. Highly negatively charged and positively charged variants (TS-14, TS-11, and TS + 5) displayed an elevated systemic clearance compared to variants with a more moderate negative charge (TS-8, TS-4) and the wild-type TS. Furthermore, whole-body pharmacokinetic analyses demonstrated a markedly enhanced accumulation across all major organs for TS + 5 compared to the wild-type mAb (TS) and TS-8. These observations of charge impacting the non-specific uptake and distribution of IgG might be relevant to ADCs, and the modification of the net surface charge might alter the non-specific uptake of ADCs in healthy tissues and subsequently influence their toxicity. Indeed, Zhao et al. demonstrated that altering the charge of an MMAF-conjugated ADC via the attachment of polylysine positively charged peptides or polyglutamate negatively charged peptides led to changes in the non-specific cellular uptake of the ADC in human primary corneal epithelial cells and affected its cellular cytotoxicity [40].

#### 2.1.2. Off-Target Receptor-Mediated Uptake of ADCs

As a part of the immune system, IgGs communicate with different immune cell types via the interaction of the fragment crystallizable (Fc) domain with Fc receptors expressed on the surface of the immune cells [41]. One of the major classes of Fc receptors that interact with IgG is the Fc gamma receptor (FcγR). These communications activate several IgG-mediated effector immune functions against the target; however, binding to the Fc domain may potentially lead to the target-independent uptake and toxicity of ADCs in immune cells [32]. Uppal et al. suggested that FcγRs might contribute to the frequent occurrence of thrombocytopenia associated with trastuzumab emtansine (T-DM1) treatment [42]. Thrombocytopenia was considered to be a target-independent toxicity, as platelets and platelet-forming megakaryocytes (MKs) have no expression of HER-2, the target of T-DM1 [32]. This study demonstrated that T-DM1 had minimal effects on mature MKs while being internalized and exhibiting potent cytotoxicity in differentiating MKs derived from human bone marrow. The off-target toxicity appeared to be mediated through FcγRIIa, and blocking FcγRIIa binding inhibited T-DM1 uptake. However, in another study, Zhao et al. showed that the uptake of T-DM1 in differentiating MKs was independent of FcγRIIa, but instead mediated by macropinocytosis [43]. In agreement with results from Zhao et al., a recent study by Aoyama et al. indicated that blocking FcγRIIa did not affect the cellular uptake and cytotoxicity of monomeric ADCs in MEG01-S, a FcγRIIa-expressing human megakaryoblastic leukemia cell line [44]. Instead, they observed that ADC aggregates activated FcγRs, leading to an increased uptake and cytotoxicity in FcγRs-expressing cells but not in FcγRs-negative cells. These results align with previous studies that showed enhanced activation of FcγRs by mAb aggregates, which potentially leads to the higher internalization and lysosomal degradation of mAb aggregates in immune cells compared to native mAbs [45,46]. These findings are particularly relevant for the first-generation ADCs, where aggregates commonly developed; aggregation issues have been minimized in later generation ADCs due to the optimization of several factors, including the linker/payload, DAR, and conjugation chemistries [47,48,49]. In addition to megakaryocytes, macrophages also exhibit a high expression level of FcγRs, which might render them susceptible to ADC off-target toxicity via Fc-mediated uptake [50]. Interstitial lung disease (ILD)/pneumonitis is one of the prevalent life-threatening adverse events associated with anti-HER2 ADC therapies including trastuzumab emtansine, trastuzumab deruxtecan, and trastuzumab doucamazine [51]; however, the underlying cause of ADC-induced ILD is still poorly understood. In a recently published study in monkeys, Kumagai et al. demonstrated a significant distribution of trastuzumab deruxtecan in alveolar macrophages, which localize within the alveolar space where ADC-induced pathological lesions occur [52]. Given that alveolar macrophages express high amounts of FcγR [50,53], and given that respiratory alveoli exhibit a low expression of HER2 [54], Fc-mediated non-specific uptake might contribute to ADC-induced ILD. Investigations with engineered ADCs with ablated FcγR binding might help to define the role of FcγR in ADC-induced ILD. 

Besides FcγRs, mannose receptor (MR) binding and receptor-mediated internalization has been proposed as a potential mechanism that mediates the off-target hepatic toxicity of ADCs [55]. MR, which belong to the C-type lectin receptor family, are expressed widely in various tissues and play an essential role in recycling glycosylated endogenous and exogenous proteins [32]. Receptor-mediated uptake by MR is one of the critical clearance mechanisms of biotherapeutic proteins. For example, the systemic clearance of therapeutic IgGs with a high level of mannosylation is significantly faster than that of normal IgGs in humans [56]. Kogelberg et al. demonstrated MR-mediated uptake of MFECP1, a mannosylated antibody-enzyme fusion protein, by liver sinusoidal endothelial cells (SECs). Marked inhibition of MFECP1 clearance was observed with the co-administration of the MR inhibitor mannan [57]. Hepatic adverse events are frequently reported as target-independent toxicities of several ADCs [12]. In addition, acute thrombocytopenia and sinusoidal obstruction syndrome (SOS) related to ADC treatment have been linked to the degeneration and loss of liver SECs [58]. Therefore, MR-mediated uptake by liver SECs is a possible contributor to the target-independent hepatic toxicity of ADCs.

### 2.2. Off-Site, On-Target Toxicity

Even though evidence suggests that payload-mediated off-target mechanisms drive the majority of ADC toxicities, the binding of ADCs to target antigens expressed in healthy tissues could also lead to significant toxicities [59]. For example, around 40% of patients treated with enfortumab vedotin in clinical trials experienced dysgeusia [60], which is considered to be an on-target toxicity due to the expression of the ADC target (nectin-4) in the salivary glands [61]. Indeed, this toxicity is uncommon in patients treated with other approved MMAE-ADCs, including brentuximab vedotin, polatuzumab vedotin, and tisotumab vedotin [62,63,64]. As shown in the example above, ADCs producing toxicity that is not typically associated with their payload may be suggestive of an on-target mechanism. The converse is also true; the observation of a common toxicity for several ADCs targeting the same antigen with different payloads is also suggestive of an on-target mechanism. For instance, ILD and pneumonitis were identified as adverse events leading to the dose modification, dose delay, or treatment discontinuation of the anti-HER2 ADC trastuzumab deruxtecan in the phase 2 DESTINY-Breast01 clinical trial [65]. These same toxicities, severe and lethal cases of ILD and pneumonitis, also occurred in patients treated with additional anti-HER2 ADCs, including trastuzumab duocarmazine and trastuzumab emtansine [66,67]. In addition, serious cardiac toxicity, including a decrease in left ventricular ejection fraction (LVEF), has been observed in patients treated with the trastuzumab-based ADCs mentioned above. These toxicities are also included in the black box warning for trastuzumab treatment [55], suggesting that on-target uptake of the ADC, rather than the non-specific, off-target uptake of payload, is the primary driver of toxicity. 

Interestingly, the application of the same ADC to treat different cancers may lead to different toxicities [8]. For example, severe rash was one of the dose-limiting toxicities of glembatumumab vedotin, which might be an on-target toxicity relating to the expression of its target, gpNMB, in the skin [68,69]. Following the administration of the same dose of glembatumumab vedotin, grade ≥ 3 rash occurred in 4% of patients with advanced breast cancer but in 30% of patients with advanced myeloma [69,70]. Pruritus and alopecia also occurred more frequently in melanoma patients than in breast cancer patients (63% and 65% vs. 21% and 25%, respectively). The mechanism behind this phenomenon is still unknown, but it is possibly related to the effects of the two types of cancer on the expression of gpNMB by healthy cells. In another example, LOP628, an anti-KIT-SMCC-DM1 ADC, caused life-threatening rapid hypersensitivity reactions (HSR) in several patients, which led to the termination of its clinical development [71]. Data from L’Italien et al. implicate that mast cell degranulation due to co-engagement of KIT and FcγRs by the parental anti-KIT mAb is the leading cause of HSR [71]. This particular case presents a unique mechanism in which the on-target toxicity does not involve cytotoxicity to the antigen-expressing cells but rather works by activating the immune system through a costimulatory signaling pathway. Additionally, the target antigen expression in healthy tissues does not always lead to on-target toxicity. For instance, the expression of the membrane-associated mucin MUC16 was reported in the human ocular surface epithelia; nevertheless, no ocular toxicity occurred in patients treated with DMUC5754A, an anti-MUC16 MMAE-ADC, in clinical trials [72,73]. Another antigen, TROP-2, is known to be expressed widely in various tissues [74]; however, the clinical toxicity profile of sacituzumab govitecan, an anti-TROP2 SN-38 conjugated ADC, largely resembles that of the SN-38 payload, suggestive of off-target mechanisms [59]. The low on-target toxicity of sacituzumab govitecan might relate to: (1) the limited accessibility of the antigen in non-malignant tissues compared to tumors; (2) an insufficient expression level to induce toxicities; and (3) the lower sensitivity of normal tissues to the SN-38 payload compared to cancerous tissues [75]. In addition, since sacituzumab govitecan employs a pH-sensitive linker engineered to release the payload within an acidic environment, the higher pH found within the extracellular fluid of normal tissues relative to tumors might contribute to the low on-target toxicity observed for this ADC [76]. 

## 3. Clinical Toxicity Profiles of Antibody-Drug Conjugates

### 3.1. Approved ADCs

In this section, the clinical toxicity profiles of the approved ADCs are discussed in detail. A summary of these information can be found in Table 1.

#### 3.1.1. ADCs with Calicheamicin Payload

##### Gemtuzumab Ozogamicin (Mylotarg)

Gemtuzumab ozogamicin consists of a humanized anti-CD33 IgG linked to a DNA-alkylating calicheamicin payload via a pH-sensitive hydrazone linker [77,78]. In the first phase 1 dose-escalation study, 40 patients with relapsed or refractory CD33-positive acute myeloid leukemia (AML) were treated with 0.25 mg/m^2^ to 9 mg/m^2^ of gemtuzumab ozogamicin [79]. The dose of 9 mg/m^2^ was selected for the subsequent phase 2 study because saturation of the CD33 binding sites, regardless of the leukemic burden, was observed at this dose level. In the phase 2 study, 142 patients with first relapse CD33-positive AML were treated with 9 mg/m^2^ of gemtuzumab ozogamicin once every two weeks [80]. The most common adverse events of all grades (≥30%) included thrombocytopenia, fatigue, neutropenia, pyrexia, nausea, infection, chills, hemorrhage, vomiting, headache, stomatitis, diarrhea, and abdominal pain. Most of the treated patients experienced grade 3 or 4 neutropenia (97%) and thrombocytopenia (99%). Other grade ≥3 treatment-related adverse events included increases in AST or ALT levels (17%), sepsis (16%), fever (15%), chills (13%), nausea and vomiting (11%), dyspnea (9%), hypertension (9%), hypotension (8%), pneumonia (7%), and asthenia (7%). The clinically relevant serious adverse events were neutropenia (34.3%), thrombocytopenia (21.7%), and infusion-related reactions (2.5%). The most common causes of treatment discontinuation were infection, hemorrhage, multi-organ failure, and veno-occlusive disease/sinusoidal obstruction syndrome (VOD/SOS). 

Gemtuzumab ozogamicin was approved in 2001 by the FDA; however, post-marketing studies revealed significant systemic toxicities and poor efficacy [81]. In phase 3 randomized comparative trial SWOGS0106, 637 AML patients were treated with either gemtuzumab ozogamicin in combination with other chemotherapeutic agents, including daunorubicin and cytosine arabinoside, or chemotherapy alone [82]. While the combination therapy with gemtuzumab ozogamicin showed no significant clinical benefit, a marked increase in the mortality rate due to toxicity was observed in the combination arm (5.7%) compared to the chemotherapy arm (1.4%). Based on these results, gemtuzumab ozogamicin was voluntarily withdrawn in June 2010. Subsequently, several clinical studies at lower recommended doses and a different dosing schedule demonstrated improved clinical outcomes and more favorable toxicity profiles, which led to the re-approval of gemtuzumab ozogamicin in 2017 [83,84,85]. 

Hepatotoxicity, including life-threatening and sometimes fatal hepatic VOD events in patients receiving gemtuzumab ozogamicin as a single agent or as part of a combination chemotherapy regimen, is included in the black box warning [86]. In phase 2 clinical trials conducted in 277 AML patients, VOD occurred in 5% of the patients and caused fatal reactions in 3% [87,88]. The expression of CD33 in hepatocytes is likely the leading cause of gemtuzumab ozogamicin-mediated hepatotoxicity [89,90]. In addition, clinical trials of the anti-CD33 ADC vadastuximab talirine (SGN-CD33A) were put on hold in December 2016 due to the induction of VOD/SOS in clinical settings which resulted in the death of four out of six SOS cases [91]. Besides hepatocytes, CD33 is also highly expressed in hematopoietic cells, leading to the significant hematologic toxicities of anti-CD33 therapies [92,93].

##### Inotuzumab Ozogamicin (Besponsa)

Inotuzumab ozogamicin consists of a humanized anti-CD22 IgG linked to a calicheamicin payload via a pH-sensitive hydrazone linker [94]. In a phase 1/2 dose-escalation and dose-expansion study, relapsed/refractory acute lymphoblastic leukemia patients were treated with inotuzumab ozogamicin at doses of 1.2 mg/kg to 1.8 mg/kg per 28-day cycle [95]. Neutropenia and thrombocytopenia were the most common treatment-related adverse events. There were four reported cases of VOD/SOS, including one fatal case. The dose of 1.8 mg/m^2^ (0.8 mg/m^2^ on day 1; 0.5 mg/m^2^ on days 8 and 15) was selected for the subsequent phase 3 clinical trial. 

In a phase 3 randomized open-label INO-VATE ALL study, patients with relapsed/refractory acute lymphoblastic leukemia were treated with either 1.8 mg/m^2^ of inotuzumab ozogamicin per cycle (n = 139) or standard chemotherapy (n = 120) [96]. Among the patients treated with inotuzumab ozogamicin, the most common (≥15%) treatment-related adverse events of all grades were neutropenia (36%), thrombocytopenia (29%), infection (48%), anemia (18%), leukopenia (17%), febrile neutropenia (16%), and nausea (15%). The grade ≥3 treatment-related adverse events most commonly reported (≥10%) were neutropenia (34%), thrombocytopenia (20%), leukopenia (15%), febrile neutropenia (14%), anemia (11%), and lymphopenia (11%). Fatal infections, including pneumonia, neutropenic sepsis, sepsis, septic shock, and pseudomonal sepsis, were reported in 5% of patients. Dose reductions and discontinuation due to adverse events occurred in 2% and 9% of patients. Cases of VOD/SOS occurred in 14% of patients, including five fatal cases (3%) [97]. Patients who underwent hematopoietic stem cell transplants after inotuzumab ozogamicin treatment have an increased risk of VOD. As a result, hepatotoxicity is included in the black box warning for patients who received inotuzumab ozogamicin [98]. Since CD22 is not expressed in the liver, the target-independent uptake mechanisms of either the ADC or the free payload play a potential role in hepatotoxicity [91].

#### 3.1.2. ADCs with Auristatin Payloads

##### Brentuximab Vedotin (Adcetris)

Brentuximab vedotin is a CD30-targeting chimeric IgG1 conjugated to MMAE via a protease cleavable valine-citrulline (vc) linker [99]. The first phase I clinical study of this ADC was conducted in 45 relapsed or refractory patients with CD30-positive hematologic malignancies, including Hodgkin’s lymphoma (HL), anaplastic large-cell lymphoma (ALCL), and angioimmunoblastic T-cell lymphoma [100]. A traditional 3 + 3 dose-escalation study design was performed with intravenous infusion of brentuximab vedotin at doses of 0.1 to 3.6 mg/kg every three weeks as salvage therapy. A single patient who received the 3.6 mg/kg dose experienced febrile neutropenia leading to sepsis and death two weeks after dosing. Dose-limiting toxicities were observed in several patients treated with the 2.7 mg/kg dose, including grade 3 hyperglycemia, unrelated grade 3 acute renal failure, and unrelated grade 3 prostatitis and febrile neutropenia. Only one patient in the 1.8 mg/kg dose cohort experienced dose-limiting toxicity (grade 4 thrombocytopenia). Based on this observation, the dose of 1.8 mg/kg was identified as the maximum tolerated dose. The most common adverse effects observed in this trial were peripheral neuropathy, neutropenia, pyrexia, diarrhea, and nausea.

In the pivotal phase II trial, 102 patients with relapsed or refractory HL after autologous stem cell transplantation were treated with 1.8 mg/kg of brentuximab vedotin by IV infusion every three weeks as salvage therapy [101]. The most common therapeutic-related adverse events of any grade were peripheral sensory neuropathy (42%), nausea (35%), fatigue (34%), neutropenia (19%), diarrhea (18%), pyrexia (14%), vomiting (13%), arthralgia (12%), pruritus (12%), myalgia (11%), peripheral motor neuropathy (11%), and alopecia (10%). Serious adverse events of grade 3 and 4 severity were observed in 55% of patients, including neutropenia (20%), peripheral sensory neuropathy (8%), thrombocytopenia (8%), and anemia (6%). Treatment discontinuation due to adverse events occurred in 20% of patients, with peripheral sensory neuropathy (6%) and peripheral motor neuropathy (3%) being the most common events. Dose delays occurred in 8% of patients, with neutropenia (16%) and peripheral sensory neuropathy (13%) being the most common causes. Dose reductions from 1.8 to 1.2 mg/kg were required in 11 patients, mostly due to peripheral neuropathy (10 of 11 patients). A similar toxicity profile was observed in another phase II clinical trial for patients with relapsed or refractory systemic anaplastic large-cell lymphoma, with additional common grade 1 or 2 adverse effects including rash (24%), constipation (22%), headache (19%), cough (17%), dyspnea (17%), upper respiratory tract infection (17%), decreased appetite (16%), dizziness (16%), insomnia (16%), chills (14%), muscle spasms (14%), thrombocytopenia (14%), weight loss (14%), edema peripheral (12%), and pain in extremity (12%) [102]. 

In the AETHERA study, a randomized, double-blind, placebo-controlled phase III clinical trial, patients with Hodgkin’s lymphoma who were at risk of relapse or progression after autologous stem cell transplantation were treated with brentuximab vedotin as consolidation therapy [103]. The most common adverse events at any grade occurring in the brentuximab vedotin-treated group at a significantly higher incidence compared to the placebo group were peripheral sensory neuropathy (56% vs. 16%), neutropenia (35% vs. 12%), and peripheral motor neuropathy (23% vs. 2%). Severe adverse events at grade ≥3 also included peripheral sensory neuropathy (10% vs. 1%), neutropenia (29% vs. 10%), and peripheral motor neuropathy (6% vs. 1%). The incidences of treatment discontinuation and dose modifications (dose reduction or delay) due to peripheral neuropathy were 23% and 31%, respectively. Neutropenia caused dose delays in 22% of patients. 

During post-marketing surveillance, several fatal cases of progressive multifocal leukoencephalopathy resulting from John Cunningham virus infection have been reported. Therefore, the black box warning for brentuximab vedotin has included this potential risk [62]. Other severe and fatal adverse events associated with brentuximab vedotin include febrile neutropenia, hepatotoxicity, pneumonitis, interstitial lung disease, acute respiratory distress syndrome, Stevens-Johnson syndrome, toxic epidermal necrolysis, acute pancreatitis, perforation, hemorrhage, erosion, ulcer, intestinal obstruction, enterocolitis, neutropenic colitis, and ileus. 

##### Polatuzumab Vedotin (Polivy)

Polatuzumab Vedotin consists of a humanized anti-CD79b IgG linked to an MMAE payload via a protease cleavable valine-citrulline linker [104]. The first-in-human phase I clinical trial of polatuzumab vedotin was conducted in two dose-escalation cohorts, relapsed/refractory non-Hodgkin’s lymphoma (NHL) patients (n = 34) and chronic lymphocytic leukemia (CLL) patients (n = 18) [19]. In the NHL cohort, patients were treated with polatuzumab vedotin at 0.1 to 2.4 mg/kg doses every three weeks. Dose-limiting toxicity (grade 4 neutropenia) was observed in one of the ten patients (10%) treated at a 2.4 mg/kg dose, the recommended dose for the subsequent phase 2 study. In the CLL cohort, patients were treated with polatuzumab vedotin at 0.25 to 1.8 mg/kg doses. Dose-limiting adverse events (grade 4 neutropenia and grade 4 fungal infection) occurred in two of the five patients (40%) treated at a 1.8 mg/kg dose. None of the 18 patients with CLL achieved objective responses. Another two dose-expansion NHL cohorts received either 2.4 mg/kg polatuzumab vedotin alone (n = 45) or in combination with rituximab (n = 9). Among the 45 NHL patients treated with polatuzumab vedotin at the recommended phase 2 dose of 2.4 mg/kg, 26 (58%) experienced at least one grade 3–4 adverse event, with the most common adverse events reported in more than two patients including neutropenia (40%), anemia (11%), and peripheral neuropathy (9%). Treatment discontinuation due to adverse events occurred in 23 (51%) patients, with peripheral sensory neuropathy being the most common cause (11 of 45 patients). A treatment delay of at least one dose occurred in 17 (38%) patients, with neutropenia being the most common reason (11 of 45 patients). A dose reduction to 1.8 mg/kg occurred in six (13%) patients due to neutropenia (two patients), sensory neuropathy (two patients), paranesthesia (one patient), and diarrhea (one patient). Among the nine patients with NHL treated with the combination therapy of polatuzumab vedotin and rituximab, grade 3–4 adverse events occurred in seven patients (77%), with neutropenia (five patients), anemia (two patients), and febrile neutropenia (two patients) being the most common.

In the ROMULUS phase 2 clinical trial, patients with relapsed/refractory diffuse large B-cell lymphoma (DLBCL, n = 81) or follicular lymphoma (FL, n = 41) were randomized to receive 375 mg/m^2^ of rituximab plus 2.4 mg/kg of either polatuzumab vedotin (R-pola) or pinatuzumab vedotin (R-pina) [105]. Pinatuzumab vedotin is an investigational anti-CD22-MMAE ADC also developed by Genentech. The most common treatment-related adverse events reported for both treatments were diarrhea, fatigue, peripheral neuropathy, nausea, and neutropenia. Among patients treated with R-pola, the most common grade 3–4 adverse events in the DLBCL cohort were neutropenia (23%), anemia (8%), and diarrhea (8%), and those in the FL cohort were neutropenia (15%) and diarrhea (10%). Peripheral neuropathy occurred in more than half of the patients treated with R-pola and resulted in treatment discontinuations in 18% and 55% of the patients in the DLBCL and FL cohorts, respectively. Consequently, the maximal dose of polatuzumab vedotin in combination therapy used for the subsequent dose-escalation clinical trial was reduced to 1.8 mg/kg. 

The pivotal GO29365 phase 1b/2 clinical trial was conducted to evaluate the safety and efficacy of the combination treatments of polatuzumab vedotin (1.8 mg/kg) with bedamustine plus either obinutuzumab (pola-BG) or rituximab (pola-BR) in transplantation-ineligible relapsed/refractory DLBCL [106]. An initial safety phase 1b included six patients treated with pola-BR and six patients treated with pola-BG, followed by a phase 2 including an expansion cohort treated with pola-BG (n = 21) and a cohort (n = 80) randomly assigned to treatments of either pola-BR or BR alone. Among the cohort with treatment randomized to BR or pola-BR, the grade 3–4 adverse events that were more common with the pola-BR treatment were anemia (28% vs. 18%), thrombocytopenia (41% vs. 23%), and neutropenia (46% vs. 33%). Peripheral neutropenia and diarrhea of all grades were more prevalent with pola-BR (44% and 39% vs. 3% and 28%, respectively). A polatuzumab vedotin dose reduction occurred in 5% of the patient solely due to peripheral neuropathy, and treatment discontinuation due to adverse events was more common with the pola-BR treatment (33% vs. 10%). Similar results were observed in another clinical trial in the Japanese population [107]. 

##### Enfortumab Vedotin (Padcev)

Enfortumab vedotin is composed of a fully human anti-nectin-4 IgG conjugated to an MMAE payload via a protease cleavable valine-citrulline linker [61]. The phase 1 EV-101 dose-escalation/dose-expansion study was conducted in patients with nectin-4-positive solid tumors [20]. This study consisted of three parts: (A) to establish the maximum tolerated dose and the recommended phase 2 dose of enfortumab vedotin; (B) to evaluate enfortumab vedotin in three dose-expansion cohorts, including patients with metastatic urothelial carcinomas (mUC) and severe renal insufficiency, patients with non-small-cell lung cancer, and patients with ovarian cancer; (C) to evaluate enfortumab vedotin in a dose-expansion cohort in mUC patients previously treated with an immune checkpoint inhibitor. The recommended phase 2 dose was determined to be 1.25 mg/kg. Among the 155 mUC patients, the most common adverse events of any grade were fatigue (53%), alopecia (46%), decreased appetite (42%), dysgeusia (38%), nausea (38%), peripheral sensory neuropathy (38%), pruritus (35%), diarrhea (33%), and maculopapular rash (27%). A similar toxicity profile was observed in the EV-201 phase II clinical trial, which consisted of a single-arm study of enfortumab vedotin 1.25 mg/kg in 125 patients with advanced or metastatic UC who were previously treated with platinum chemotherapy or immune checkpoint inhibitors [108]. Treatment-related grade 3–4 adverse events occurred in 54% of the patients, with rash (17%), neutropenia (8%), anemia (7%), and fatigue (6%) being the most common.

In the two-arm phase 3 EV-301 clinical trial, patients with advanced or metastatic UC who had previously received platinum chemotherapy or immune checkpoint inhibitors were treated with either enfortumab vedotin (1.25 mg/kg, n = 296) or chemotherapy (n = 291) [109]. In the cohort treated with enfortumab vedotin, the most common grade ≥3 adverse events (≥5%) were maculopapular rash (7.4%), fatigue (6.4%), and decreased neutrophil count (6.1%). Dose delays occurred in 61% of patients, with peripheral neuropathy (23%), rash (11%), and fatigue (9%) being the most common reasons (≥4%). Dose reductions occurred in 34% of patients, with peripheral neuropathy (10%), rash (8%), decreased appetite (3%), and fatigue (3%) being the most common reasons (≥2%). Treatment discontinuation occurred in 17% of patients, with peripheral neuropathy (5%) and rash (4%) being the most common reasons (≥2%).

Severe skin reactions are included in the black box warning for enfortumab vedotin [60]. Treatment-related skin reactions, which occurred in 55% of the patients treated with enfortumab vedotin in clinical trials, were expected as on-target off-tumor toxicities due to nectin-4 expression in the skin [110]. Grade ≥3 skin reactions occurred in 13% of patients, leading to dose interruption/reduction in 9% of patients and treatment discontinuation in 2.6% of patients [60]. Severe cutaneous adverse reactions, including fatal Stevens-Johnson syndrome and toxic epidermal necrolysis, were reported during clinical trials and post-marketing settings [110]. Skin adverse reactions were also common in patients treated with other ADCs utilizing an MMAE payload, including brentuximab vedotin (31%), glembatumumab vedotin (44%), and polatuzumab vedotin (13–31%), suggesting a potential contribution of MMAE to skin toxicities [62,63,111,112]. Hyperglycemia and pneumonitis are also included in the warning box due to several life-threatening or fatal reactions observed during clinical trials [60]. In clinical trials, hyperglycemia of any grade occurred in 14% of patients treated with enfortumab vedotin, with 7% of patients developed grade 3–4 hyperglycemia. Discontinuation of treatment due to hyperglycemia occurred in 0.6% of patients. Pneumonitis of any grade occurred in 3.1% of the patients, and discontinuation of the treatment due to grade 3–4 pneumonitis occurred in 0.7% of the patients. 

##### Tisotumab Vedotin (Tivdak)

Tisotumab vedotin is a conjugate of a human anti-tissue factor monoclonal antibody and an MMAE payload via a protease cleavable valine-citruline linker [113]. In the dose-escalation phase of the first-in-human phase 1/2 InnovaTV 201 study, 27 patients with a diverse array of advanced or metastatic solid tumors, including ovary, cervix, endometrium, bladder, prostate, esophagus, non-small-cell lung cancer, or squamous cell carcinoma of the head and neck, were treated with 0.3 mg/kg to 2.2 mg/kg of tisotumab vedotin once every three weeks [21]. Three patients in the 2.2 mg/kg dose cohort had dose-limiting toxicities, including type 2 diabetes mellitus, mucositis, and neutropenic fever. Therefore, the maximum tolerated dose and recommended phase 2 dose was determined as 2.0 mg/kg. In the dose-expansion phase of this study, 147 patients with cancer of the ovary, cervix, endometrium, bladder, prostate, and esophagus, as well as non-small-cell lung cancer, were treated with 2.0 mg/kg of tisotumab vedotin once every three weeks. Across tumor types in phase 2, the most common (≥20%) treatment-related adverse events of any grade were epistaxis (69%), fatigue (56%), nausea (52%), alopecia (44%), conjunctivitis (43%), decreased appetite (36%), constipation (35%), diarrhea (30%), vomiting (29%), peripheral neuropathy (22%), dry eye (22%), and abdominal pain (20%). The most common (>2%) treatment-related adverse events of grade 3 or worse were fatigue (10%), anemia (5%), abdominal pain (4%), hypokalemia (4%), conjunctivitis (3%), hyponatremia (3%), and vomiting (3%).

In the phase 2 open-label single-arm InnovaTV204 study, 101 patients with recurrent or metastatic cervical cancer with disease progression on or after chemotherapy were treated with 2.0 mg/kg of tisotumab vedotin every three weeks until disease progression or unacceptable toxicity [114]. The most common (≥25%) adverse reactions, including laboratory abnormalities, were decreased hemoglobin (59%), fatigue (57%), a decrease in lymphocytes (50%), nausea (41%), peripheral neuropathy (46%), alopecia ((39%), epistaxis (39%), conjunctival adverse reactions (37%), hemorrhage (38%), a decrease in leukocytes (30%), increased creatinine, dry eye (29%), an increased prothrombin international normalized ratio (26%), a prolonged activated partial thromboplastin time (26%), diarrhea (27%), and rash (25%) [64]. Serious adverse reactions occurred in 43% of patients, with ileus (6%), hemorrhage (5%), pneumonia (4%), peripheral neuropathy (3%), sepsis (3%), constipation (3%), and pyrexia (3%) being the most common. A dose interruption occurred in 47% of patients, with peripheral neuropathy (8%), conjunctival adverse reactions (4%), and hemorrhage (4%) being the most common reasons. A dose reduction occurred in 23% of patients, with conjunctival adverse reactions (9%) and corneal adverse reactions (8%) being the most common reasons. Treatment discontinuation occurred in 13% of patients, with peripheral neuropathy (5%) and corneal adverse reactions (4%) being the most common reasons. 

Ocular toxicity is included in the black box warning for patients treated with tisotumab vedotin [64]. Immunohistochemical evaluation in normal human ocular tissues confirmed the expression of tissue factor in the ocular epithelium, suggesting a target-mediated uptake of tisotumab vedotin in the eye is the likely cause of toxicity [115,116]. Ocular adverse reactions occurred in 60% of patients with cervical cancer across clinical trials, with conjunctival adverse reactions (40%), dry eye (29%), corneal adverse reactions (21%), and blepharitis (8%) being the most common. Grade 3 ocular adverse reactions occurred in 3.8% of patients, including severe ulcerative keratitis in 3.2% of patients. Ocular adverse reactions led to the discontinuation of tisotumab vedotin in 6% of patients with cervical cancer.

##### Belantamab Mafodotin (Blenrep)

Belantamab mafodotin consists of a humanized afucosylated Fc-engineered IgG targeting B-cell maturation antigen (BCMA) covalently linked to MMAF payload via a non-cleavable maleimidocaproyl (mc) linker. The first-in-human phase 1 DREAMM-1 clinical trial explored belantamab mafodotin as monotherapy in relapsed or refractory multiple myeloma (RRMM) [117]. This study comprised two parts: (I) a dose-escalation phase assessing the safety and tolerability of belantamab mafodotin and determining the recommended phase 2 dose; (II) a dose-expansion phase evaluating the safety and tolerability pharmacokinetics and clinical activity of the recommended phase 2 dose. In part I, 38 patients received 0.03 to 4.6 mg/kg belantamab mafodotin every three weeks. The treatment was well-tolerated with no maximal tolerated dose established. The recommended phase 2 dose was determined to be 3.4 mg/kg based on activity and safety data. In part II, the most common adverse events at any grade were blurred vision, dry eye, thrombocytopenia, anemia, increased aspartate aminotransferase, and cough. The most frequent grade 3–4 adverse events were thrombocytopenia (35%), anemia (17%), pneumonia (6%), and infusion-related reactions (6%). 

The phase 2 DREAMM-2 study assessed the safety and efficacy of belantamab mafodotin in patients with relapsed or refractory multiple myeloma via two dosage cohorts: 2.5 mg/kg (97 patients) and 3.4 mg/kg (99 patients) once every three weeks [118]. The most common adverse events (≥20%) were keratopathy occurring in 72% of patients in the 2.5 mg/kg cohort and 77% in the 3.4 mg/kg cohort, thrombocytopenia (35% and 58%), anemia (24% and 37%), nausea (24% and 32%), pyrexia (22% and 25%), blurred vision (22% and 30%), and increased aspartate aminotransferase (20% and 24%). The most common grade 3–4 adverse events were keratopathy (27% in the 2.5 mg/kg cohort and 21% in the 3.4 mg/kg cohort), thrombocytopenia (20% and 33%), and anemia (20% and 25%). Dose delays and dose reduction due to treatment-related adverse events occurred mainly due to keratopathy (47% in the 2.5 mg/kg cohort and 53% in the 3.4 mg/kg cohort) and (25% vs. 30%), respectively. Treatment discontinuation due to ocular toxicities occurred in 1% vs. 3% of patients. Due to the prevalence of ocular adverse events, topical steroid prophylaxis was utilized before the treatment of belantamab mafodotin to prevent ocular toxicities; however, it was not proven beneficial. 

Ocular toxicity was included in the box warning in the package insert of the commercial belantamab mafodotin [119]. Ocular adverse reactions occurred in 77% of the 218 patients in the pooled safety population, including keratopathy (76%), changes in visual acuity (55%), blurred vision (27%), and dry eye (19%). Most keratopathy events developed within the first two treatment cycles (cumulative incidence of 65% by cycle 2).

Based on the results from the DREAMM-2 study, belantamab mafodotin was granted an accelerated approval by the FDA as monotherapy for patients with relapsed or refractory multiple myeloma. However, the ADC was voluntarily withdrawn from the US market in November 2022 due to its failure to meet the objective outcome of the phase 3 DREAMM-3 study [120]. Currently, belantamab mafodotin is under evaluation as combination therapy with chemotherapeutic agents for RRMM treatment in DREAMM-7 and DREAMM-8 confirmatory clinical trials. 

#### 3.1.3. ADCs with Maytansinoid Payloads

##### Trastuzumab Emtansine (Kadcyla)

Trastuzumab emtansine comprises the humanized anti-human epidermal growth factor receptor 2 (HER-2) trastuzumab, a DM1 cytotoxic payload, and an SMCC non-cleavable linker [121]. The initial phase 1 dose-escalation study assessed the safety, tolerability, and pharmacokinetics of trastuzumab emtansine in patients with locally advanced or metastatic HER2-positive breast cancer [122,123]. Two administration schedules, once every three weeks and once weekly, were evaluated. In the cohort that received treatment once every three weeks, 24 patients were treated with 0.3 to 4.8 mg/kg of trastuzumab emtansine, and the dose of 3.6 mg/kg was determined to be the maximum tolerated dose. The most common treatment-related adverse events of any grade that occurred in these patients were thrombocytopenia (54%), elevated transaminases (42%), fatigue (38%), anemia (29%), and nausea (25%). In the cohort that received trastuzumab emtansine doses of 1.2 to 2.9 mg/kg once weekly (n = 28), the maximum tolerated dose was 2.4 mg/kg. Grade ≥3 adverse events occurred in 68% of patients, of which anemia (14%), thrombocytopenia (11%), pneumonia (11%), and increased AST (11%) were the most common. 

In the phase III randomized open-label clinical study EMILIA, 991 patients with unresectable or metastatic breast cancer, who had previously been treated with trastuzumab and a taxane, were given either trastuzumab emtansine (3.6 mg/kg every three weeks) or with lapatinib plus capecitabine [124]. The most common treatment-related adverse events of any grade in the trastuzumab emtansine group were nausea (39.2%), fatigue (35.1%), and thrombocytopenia (28.0%). Grade ≥ 3 adverse events occurred in 43.1% of patients, with thrombocytopenia (14%), increased aspartate aminotransferase levels (5%), and anemia (4%) being the most frequent. Dose delays occurred in 23.7% of patients, commonly due to neutropenia, thrombocytopenia, leukopenia, fatigue, increased transaminases, and pyrexia. Dose reductions were reported in 16.3% of patients, primarily due to thrombocytopenia, increased transaminases, and peripheral neuropathy. Treatment discontinuation occurred in 6.5% of patients, with thrombocytopenia and elevated transaminases being the most common causes. 

Hepatotoxicity is included in one of the black box warnings for trastuzumab emtansine treatment, presenting as asymptomatic transient increases of serum transaminases [66]. Liver failure and fatal cases of severe treatment-related liver injury have been reported. The hepatotoxicity associated with trastuzumab emtansine treatment might be due to the HER2-dependent and independent pathways. HER2 is expressed in normal human hepatocytes, and HER-2 mediated internalization of trastuzumab emtansine causes cell-cycle arrest and apoptosis in human hepatocytes in vitro [125,126]. In addition, DM1/DM4 payloads have been shown to mediate the internalization of maytansinoid-conjugated ADCs via binding to cytoskeleton-associated protein 5 (CKAP5) [127,128]. Receptor-mediated uptake of the ADC by FcγRs and mannose receptors may also contribute to liver toxicity [32]. Besides hepatotoxicity, cardiac toxicity and embryo-fetal toxicity are also included in the black box warning. Cardiac toxicity manifests as a decrease in left ventricular ejection fraction (LVEF) to less than 40%, which is related to the trastuzumab component [129]. Moreover, several complications, including oligohydramnios and fetal/neonatal death, have been associated with trastuzumab exposure during the second or third trimester of pregnancy [11,130,131].

##### Mirvetuximab Soravtansine (Elahere)

Mirvetuximab soravtansine is an ADC composed of a humanized antibody targeting the folate receptor alpha (FRα), a disulfide cleavable linker, and a DM4 cytotoxic payload [132]. In the phase 1 dose-escalation, 44 patients with advanced solid tumors were treated with mirvertuximab soravtansine at doses between 0.15 mg/kg and 7 mg/kg based on total body weight (TBW) once every three weeks [133]. At the 7 mg/kg dose level, 1 of 5 patients had a DLT of punctate keratitis. At the 5 mg/kg dose level in 11 patients, grade 3 hypophosphatemia and additional ocular toxicities were observed. Subsequently, dosing was modified based on adjusted ideal body weight (AIBW) rather than TBW, and no DLT was observed in patients given up to 6 mg/kg AIBW, which was determined to be the recommended phase 2 dose. 

In the phase 3 open-label randomized clinical trial FORWARD I, 366 patients with FRα-positive platinum-resistant ovarian cancer were given either mirvetuximab soravtansine (n = 243) at a 6 mg/kg AIBW dose or chemotherapy (n = 109) [134]. In the mirvetuximab soravtansine group, the most common treatment-related adverse events were nausea (45.7%), blurred vision (42.0%), keratopathy (32.5%), diarrhea (31.3%), fatigue (28.8%), peripheral neuropathy (26.7%), dry eye (25.9%), and decreased visual acuity (19.3%). Incidents of grade ≥ 3 treatment-related adverse events were low, with blurred vision (2.5%), peripheral neuropathy (2.5%), and diarrhea (2.1%) being the most common. Dose delays/reductions occurred in 34.3% of patients, with ocular toxicity being the most common cause (19.8%). Dose discontinuations occurred in 4.5% of patients.

#### 3.1.4. ADCs with Camptothecin Payloads

##### Trastuzumab Deruxtecan (Enhertu)

Trastuzumab deruxtecan is a humanized anti-HER2 antibody linked to deruxtecan, a camptothecin derivative, via a stable linker [135]. In the first phase 1 dose-escalation study, 22 patients with HER-2 positive advanced or metastatic breast cancer, gastric cancer, or other HER-2 expressing solid tumors were treated with 0.8 mg/kg to 8.0 mg/kg of trastuzumab deruxtecan once every three weeks [136]. No dose-limiting toxicity was observed, and MTD was not reached. Target drug exposure was achieved at the dose of 6.4 mg/kg, which was selected as the recommended phase 2 dose. 

The pivotal single-arm phase 2 DESTINY-Breast01 clinical trial consisted of two parts [65]. In the first part, advanced/metastatic breast cancer patients previously treated with more than 2 anti-HER2 therapies were randomized to receive trastuzumab deruxtecan at doses of 5.4 mg/kg (n = 50), 6.4 mg/kg (n = 48), or 7.4 mg/kg (n = 21) once every 3 weeks. In the second part, 134 patients were treated with 5.4 mg/kg doses based on the obtained efficacy and toxicity data from part 1. Among the 184 patients treated with trastuzumab deruxtecan at 5.4 mg/kg, the most common adverse events of all grades (≥20%) were nausea (77.5%), fatigue (49.8%), alopecia (49.8%), vomiting (44.3%), neutropenia (40.3%), constipation (37.5%), anemia (33.6%), decreased appetite (33.2%), diarrhea (29.2%), leukopenia (26.9%), and thrombocytopenia (24.9%). Grade ≥3 adverse events occurred in 57.1% of the patients, with neutropenia (20.7%), anemia (8.7%), nausea (7.6%), leukopenia (6.5%), lymphopenia (6.5%), and fatigue (6.0%) being the most prevalent. Dose interruption, dose reduction, and treatment discontinuation due to adverse events occurred in 35.3%, 23.4%, and 15.2% of patients, respectively, with pneumonitis (in 11 patients) and interstitial lung disease (in 5 patients) being the most common reasons. Interstitial lung disease (ILD) and pneumonitis are included in the black box warning for patients treated with trastuzumab deruxtecan [137]. Treatment-related interstitial lung disease and fatal outcomes occurred in 9% and 2.6% of patients treated with trastuzumab deruxtecan, respectively. Similar to other HER-2 targeting ADCs, patients treated with trastuzumab deruxtecan also have increased risks of embryo-fetal toxicity and left ventricular dysfunction. 

##### Sacituzumab Govitecan (Trodelvy)

Sacituzumab govitecan is a humanized anti-TROP-2 IgG linked to the active metabolite of irinotecan (SN-38) via a pH-sensitive linker [138]. In phase 1 of the first-in-human dose-escalation dose-expansion phase 1/2 study, 25 patients with diverse metastatic solid tumors were treated with 8 mg/kg to 18 mg/kg of sacituzumab govitecan on days 1 and 8 of 21-day cycles [139]. The MTD was determined to be 12 mg/kg for the first cycle, with neutropenia being the dose-limiting toxicity. However, this dose level is too toxic for subsequent cycles; therefore, the 8 mg/kg and 10 mg/kg doses were selected for the phase 2 study. In phase 2 of this study, patients with diverse metastatic epithelial cancers who had multiple prior therapies received sacituzumab govitecan at either an 8 mg/kg (n = 81) or a 10 mg/kg (n = 97) dose [140]. The most common adverse events of all grades (≥25%) reported in the 8-mg/kg and 10-mg/kg cohorts were nausea (59% vs. 63%), diarrhea (53% vs. 62%), neutropenia (42% vs. 58%), fatigue (61% vs. 52%), vomiting (36% vs. 43%), anemia (38% vs. 42%), alopecia (46% vs. 37%), and constipation (33% vs. 37%), respectively. The most common adverse events of grades ≥3 (≥10%) reported in the 8-mg/kg and 10-mg/kg cohorts were neutropenia (30% vs. 36%), anemia (13% vs. 12%), diarrhea (4% vs. 10%), and leukopenia (6% vs. 12%). Dose reductions occurred in 19% and 28% of the patients in the 8-mg/kg and 10-mg/kg cohorts, respectively. Neutropenia was the most common adverse event leading to dose delays or reductions. Significantly more patients in the 10-mg/kg cohort experienced grade ≥3 neutropenia after the first dose than in the 8-mg/kg cohort (47% vs. 21%). 

Black box warnings were added to the sacituzumab govitecan label for severe or life-threatening neutropenia and severe diarrhea [141]. These adverse events are likely mediated by released (“free”) SN-38, as the same toxicities are associated with the SN-38 prodrug ironotecan [142]. Of all patients treated with sacituzumab govitecan, neutropenia of all grades and grade ≥ 3 occurred in 61% and 47%, respectively. Febrile neutropenia occurred in 7% of patients. Diarrhea of all grades and grade ≥ 3 occurred in 65% and 12% of all patients treated sacituzumab govitecan, respectively. Neutropenic colitis occurred in 0.5% of patients.

#### 3.1.5. ADCs with Pyrrolobenzodiazepine Payloads

##### Loncastuximab Tesirine (Zynlonta)

Loncastuximab tesirine comprises a humanized anti-CD19 IgG, a valine-alanine protease cleavable linker, and a PBD DNA-alkylating cytotoxic payload [143,144]. In the dose-escalation dose-expansion phase 1 study, 183 patients with relapsed/refractory B-cell non-Hodgkin lymphoma received 15 µg/kg to 200 µg/kg of loncastuximab tesirine once every three weeks [145]. Dose-limiting toxicities included grade 4 thrombocytopenia and grade 3 febrile neutropenia, and the maximum tolerated dose was not reached; however, cumulative toxicity was observed at 200 µg/kg. The 150 µg/kg dose was selected as the recommended phase 2 dose, as it elicited encouraging responses and a lower frequency of adverse events than the 200 µg/kg dose. In addition, due to a moderate accumulation of loncastuximab tesirine, late-developing and difficult-to-manage toxicities frequently led to dose delays and dose reductions; therefore, the recommended phase 2 dose includes a planned dose reduction to 75 µg/kg after two cycles. 

In the pivotal phase 2 LOTIS-2 clinical trial, 145 patients with relapsed or refractory diffuse large B-cell lymphoma were treated with loncastuximab tesirine once every three weeks at 150 µg/kg for two cycles, and then at 75 μg/kg after that [146]. The most common treatment-related adverse events of all grades (≥25%) were neutropenia (40%), thrombocytopenia (33%), anemia (26%), fatigue (27%), and gamma-glutamyl transferase increase (40%). The most common grade ≥3 treatment-related adverse events (≥5%) were thrombocytopenia (18%), neutropenia (26%), anemia (10%), gamma-glutamyl transferase increase (16%), leukopenia (9%), lymphopenia (5%), and hypophosphatemia (6%). Serious adverse reactions occurred in 28% of patients, with febrile neutropenia, pneumonia, edema, pleural effusion, and sepsis being the most common (≥2%). Fatal adverse reactions occurred in 1% of patients due to infection. Dose delays occurred in 49% of patients, with gamma-glutamyl transferase increase, neutropenia, thrombocytopenia, and edema being the most common reasons (≥5%). Dose reductions occurred in 8% of patients, with the gamma-glutamyltransferase increase being the most common reason (≥4%). Treatment discontinuation occurred 19% of patients, due to gamma-glutamyltransferase increase, edema, and effusion (≥2%) [147].

### 3.2. Late-Stage ADCs

#### 3.2.1. Trastuzumab Duocarmazine

Trastuzumab duocarmazine consists of the anti-HER-2 trastuzumab antibody linked to a duocamycin cytotoxic payload via a hydrophilic cleavable peptide linker [148]. In the dose-escalation study of phase 1 clinical trial, 39 patients with locally advanced or metastatic solid tumors, regardless of HER-2 expression, were given trastuzumab duocarmazine at 0.3 mg/kg to 2.4 mg/kg doses every three weeks [67]. One patient treated at 2.4 mg/kg died of pneumonitis, and no other DLT was observed. The 1.2 mg/kg dose once every three weeks was recommended for subsequent studies due to promising results in patients treated at this dose level, and the 1.5 mg/kg and 1.8 mg/kg doses did not improve the benefit-to-risk ratio for patients. In the dose-expansion study, 146 patients with breast cancer (n = 99), gastric cancer (n = 17), urothelial cancer (n = 16), and endometrial cancer (n = 14) were treated at the recommended phase 2 dose of 1.2 mg/kg every three weeks. The most common treatment-related adverse events were fatigue (33%), conjunctivitis (31%), dry eye (31%), lacrimation increase (20%), decreased appetite (19%), keratitis (19%), dry skin (18%), alopecia (18%), nausea (18%), stomatitis (16%), skin hyperpigmentation (16%), and neutropenia (16%). Ocular adverse events occurred in 71% of patients, including conjunctivitis, dry eye, keratitis, blurred vision, and corneal toxicity. Grade ≥ 3 treatment-related adverse events occurred in 35% of patients, with neutropenia (6%), fatigue (4%), and conjunctivitis (3%) being the most common. Dose delays/reductions occurred in 43% of patients, and treatment discontinuations occurred in 19% of patients, mostly due to ocular toxicity (10%). Other treatment-related toxicities that led to dose discontinuations were dyspnea, decreased LVEF, and decreased appetite. 

In the phase 3 randomized TULIP trial, 437 patients with HER-2 positive locally advanced or metastatic breast cancer were treated with either trastuzumab duocarmazine (n = 291) at the dose of 1.2 mg/kg every three weeks or physician’s choice chemotherapy (n = 146) [149]. In the interim report, the most common reported adverse events for trastuzumab duocarmazine were conjunctivitis (38.2%), keratitis (38.2%), and fatigue (33.3%). Dose discontinuation occurred in 35.4% of patients treated with trastuzumab duocarmazine, mainly due to ocular toxicities (20.8%) and respiratory disorders (6.3%). 

#### 3.2.2. Disitamab Vedotin

Disitamab vedotin consists of a humanized anti-HER-2 mAb conjugated to an MMAE payload via a cleavable valine-citruline linker [150]. It was granted conditional approval in China in June 2021 and fast-track designations by the FDA in the US [151]. In the phase 1 dose-escalation study, 21 patients with HER2-overexpressed advanced solid cancers were treated with 0.1 mg/kg to 2.5 mg/kg of disitamab vedotin once every two weeks [152]. The most common treatment-related adverse events were leukopenia (61.1%), neutropenia (52.8%), fatigue (50.0%), numbness (44.4%), AST elevation (30.6%) and ALT elevation (27.8%). The maximum tolerated dose was not determined up to 2.5 mg/kg. From a pool of five phase 1–3 clinical trials in China, the most common treatment-related adverse events were leukopenia (55.4%), alopecia (54.6%), neutropenia (50.6%), increased aspartate aminotransferase (49.7%), fatigue (46.3%), increased alanine aminotransferase (42.9%), and hypoesthesia (40.9%) [151]. The most common grade ≥ 3 adverse events were neutropenia (16.9%), leukopenia (10.9%), and hypoesthesia (8.9%).

### 3.3. Frequently Reported ADC-Associated Dose-Limiting Toxicities

#### 3.3.1. Neutropenia

Neutrophils, which account for 40% to 70% of granulocytes, are an essential component of the innate immune system [153]. Neutrophils are produced in the bone marrow at a rapid rate (approximately 10^11^ cells/day) through the differentiation of hematopoietic stem cells. They have a relatively short blood circulation half-life (about one day) [154,155,156]. These unique characteristics render neutrophils more vulnerable to anti-neoplastic agents compared to other myeloid cells that have longer lifespans, including platelets (8 days) and erythrocytes (120 days) [18]. Interruption of hematopoietic cellular division in the bone marrow often results in a reduced production of neutrophils and leads to susceptibility to serious infections and infection sequela, including sepsis and febrile neutropenia. Severe neutropenia is a frequent dose-limiting toxicity associated with most MMAE-ADCs that utilize a valine-citrulline cleavable linker, including brentuximab vedotin, polatuzumab vedotin, enfortumab vedotin, and tisotumab vedotin. In addition, grade ≥ 3 neutropenia is also commonly reported for ADCs utilizing other payloads with cleavable linkers, such calicheamicin (gemtuzumab ozogamicin and inotuzumab ozogamicin), SN-38 (sacituzumab govitecan), extecan (trastuzumab deruxtecan), PBD (loncastuximab tesirine), DM4 (coltuximab ravtansine), and doucarmycine (trastuzumab duocarmazine). Neutropenia is less common in patients treated with ADCs utilizing stable linkers, including MMAF-conjugates (belantamab mafodotin) or DM1-conjugates (trastuzumab emtansine).

ADC-associated neutropenia appears to correlate with the cumulative plasma exposure to released payload (e.g., following premature release or following distribution from intracellular sites of ADC catabolism). Membrane-permeable payloads readily distribute into bone marrow and into differentiating hematopoietic cells [157]. The deconjugation of intact ADCs within extracellular fluid within the bone marrow compartment may also contribute to myelotoxicity, as differentiating neutrophils secrete serine proteases that can cleave ADC linkers [157]. In addition to the off-target mechanisms mentioned above, target-dependent mechanisms may also contribute to neutropenia for ADCs targeting leukemic antigens [77,158,159,160]. In a recent systemic analysis, Haubner et al. quantified the expression of leukemic stem cell makers. Several of them, including CD33, CD123, and CLL-1, exhibited a high expression level not only in leukemic stem cells but also in normal hematopoietic stem/progenitor cells, which are precursors for neutrophil production [161].

#### 3.3.2. Thrombocytopenia

Thrombocytopenia is a common off-target toxicity associated with ADCs that utilize stable linkers (SMCC-DM1 or mc-MMAF) and also for the potent DNA-crosslinking payload calicheamicin. For example, grade ≥ 3 thrombocytopenia occurred in 99% of acute myeloid leukemia patients [80] and 42% of acute lymphoblastic leukemia patients treated with gemtuzumab ozogamicin [98]. Grade ≥ 3 thrombocytopenia was reported in 14.5% of breast cancer patients treated with trastuzumab emtansine [66] and in 21% of multiple myeloma patients treated with belantamab mafodotin [119]. 

The definitive mechanism of thrombocytopenia remains elusive. The non-specific uptake of ADCs in differentiating megakaryocytes via either FcγR-mediated or micropinocytosis pathways was proposed as a probable cause [42,43,44]. However, based on the observation that the clinical manifestation of ADC-associated thrombocytopenia usually occurs within 24 h, which is significantly shorter than the typical platelet lifespan of 8 to 10 days, additional factors might be contributing to thrombocytopenia. Indeed, Guffroy et al. showed that monkeys treated with a calicheamicin-based ADC exhibited significant damage in the sinusoidal epithelial cells and pronounced sequestration and accumulation of platelets in the hepatic sinusoids three days after treatment, which coincided with the nadir of platelet reduction [58].

#### 3.3.3. Peripheral Neuropathy

Clinical manifestation of peripheral neuropathy includes sensory-related symptoms, such as numbness, tingling, and pain in the extremities, or, to a lesser extent, motor-related symptoms such as muscle weakness [18]. Peripheral neuropathy is a common adverse effect associated with tubulin-inhibiting chemotherapeutic agents such as taxanes and vinca alkaloids [162]. Similarly, peripheral neuropathy is a significant dose-limiting off-target toxicity associated with ADCs utilizing tubulin inhibitor payloads combined with cleavable linkers such as vc-MMAE, SPP-DM1, and SPDB-DM4 [102,134,163,164]. The mechanism behind peripheral neuropathy is hypothesized to be peripheral axonopathy induced by free payload released in the systemic circulation [18]. Even though peripheral neurons are not actively proliferating, a functional microtubule is critical for protein transport from the cell body to the distal synapses. Therefore, the disruption of microtubules by tubulin-inhibiting payloads may result in peripheral neuropathy [18].

#### 3.3.4. Ocular Toxicity

Ocular toxicity is one of the key off-target dose-limiting toxicities of ADCs containing the SPDB-DM4 linker-payload, such as cantuzumab ravtansine [165], mirvetuximab soravtansine [166], and coltuximab ravtansine [167], or the mc-MMAF linker-payload, such as belantamab mafodotin [119], AGS-16C3F [168], and SGN-75 [169]. The susceptibility of the eye to ADC cytotoxicity is possibly due to several factors, including an ample blood supply, the presence of rapidly dividing epithelial cell populations, and the high expression of several cell-surface receptors [170]. In clinical settings, symptoms of ADC-associated ocular adverse events include blurred vision, keratitis, dry eye, and microcystic epithelial damage. The mechanism underlying ocular toxicity associated with ADCs is still poorly understood. The non-specific uptake of intact ADCs via macropinocytosis was suggested by Zhao et al. as the mechanism behind the off-target ocular toxicity of ADCs [40]. However, the target-mediated uptake of ADC might also be relevant [171]. Table 2 summarizes the commonly reported dose-limiting toxicities associated with ADC treatments.

## 4. Strategies to Reduce Toxicities

The high attrition rate of ADCs containing antimitotic payloads is primarily due to a suboptimal efficacy at the MTD during clinical trials. In response, the recent development of ADCs has shown increased utilization of more potent DNA-damaging payloads such as PBD dimers, duocarmycins, and indolinobenzodiazepine dimers [24,28,172]. Unfortunately, even though these highly potent ADCs often lead to a higher efficacy, their clinical success has been limited due to the increased incidence of life-threatening toxicities. For instance, treatment with vadastuximab talirine, an anti-CD33 PBD-ADC developed by Seattle Genetics, reached a 70% complete remission rate in AML patients; however, due to the occurrence of several treatment-related fatal events, the clinical development of vadastuximab talirine has been terminated [28]. A recent analysis by Saber and Leighton from the FDA found that 47% of investigational new drug applications for PBD-conjugated ADCs between 2013 and 2017 were discontinued, mainly due to safety concerns [22]. These outcomes suggest that unwanted toxicity restricts the clinical benefit of ADCs by limiting the tolerable doses to amounts below the levels required to provide meaningful or optimal anti-cancer efficacy. In a formal comparison of effective exposure levels identified in preclinical studies with exposure profiles at the maximum tolerated ADC doses in the clinic, de Goeij and Lambert concluded that, for many ADCs, the exposures at the clinical MTDs were significantly lower than the exposures required for efficacy in preclinical models [172]. Much focus in the ADC field is shifting to strategies to widen the therapeutic window through the mitigation of ADC toxicity, as this approach, if successful, might enable increased MTDs and subsequently improved clinical outcomes. This section will discuss several strategies that have demonstrated improvements in ADC therapeutic windows and/or reductions in ADC toxicity in preclinical models or clinical settings.

### 4.1. Modifying Conjugation Technology or Drug/Linker Chemistry

The conventional conjugation of methods employing non-specific linkage of amine-based lysine or thiol-based cysteine residues of the antibody leads to considerable heterogeneity in the DAR and hydrophobicity [173]. The degree of drug loading ranges from no loading (DAR = 0) to highly-loaded ADCs (DAR ≥ 6). Highly-loaded ADCs are often unstable in plasma and exhibit high rates of non-specific uptake in the liver, leading to increased off-target toxicity [36,174]. Several approaches have been developed to precisely conjugate a designated number of cytotoxic payloads at defined antibody locations. These technologies would enable a more precise control of DAR and improve the stability of the conjugated payload due to its conjugation to more stable site, while also simplifying the in-process control and ADC manufacturing [175]. The first site-specific conjugation strategy was developed by Junutula et al. and allows the thiol-based linkage of payloads to extra cysteine residues introduced to the antibody at the Ala114 position of the CH1 domain via site-directed mutagenesis [176]. The engineered antibody, called THIOMAB, enables the site-specific coupling of MMAE payloads via a thiol-reactive linker to form THIOMAB-drug conjugates (TDCs). For instance, an anti-MUC16 TDC exhibited a similar in vivo efficacy to ADC, despite having 2-fold lower drug loading. Safety studies demonstrated that healthy rats tolerated TDC at a much higher dose (4150 µg/m^2^ MMAE, or 100 mg/kg TDC) than ADC (2250 µg/m^2^ MMAE, or 25 mg/kg ADC). In addition, reduced hematologic and hepatic toxicities were observed in rats and monkeys treated with TDC compared to ADC [176]. Since the inception of the THIOMAB technology, numerous site-specific conjugation methods have emerged, and several have reached clinical development [24,177,178]. More extensive reviews of these methods are discussed elsewhere [179,180,181,182,183,184]. 

In addition to site-specific conjugation, the modification of the linker can also reduce ADC hydrophobicity and reduce toxicity. Burke and Simmons et al. demonstrated that PEGylation of the drug-linker component increased the hydrophilicity of ADCs, which led to improved PK, tolerability, and efficacy [185,186]. They incorporated a PEG polymer with varying lengths from 2 to 24 into the ADC linker. Then, the impact of the spacer on the ADC’s in vitro potency and in vivo efficacy/tolerability was evaluated. While all ADCs with a PEG spacer had similar in vitro cellular cytotoxicity, ADCs with a PEG spacer with a length of eight or greater had slower clearance and superior in vivo anti-tumor activity. Notably, the tolerability of the ADC was significantly improved with the addition of the PEG spacer [185]. At the 50 mg/kg dose, all animals treated with non-PEGylated ADC lost more than 20% of body weight on day 6, while the animal treated with PEG_12_-ADC maintained minimal change in body weight. Further studies showed that ADCs with PEGylated linkers had reduced peak plasma and tissue concentrations, leading to substantially less hematologic toxicity [186]. In addition to MMAE, this PEGylation approach have been utilized for other payloads including maytansinoid [187,188,189], duocamycin [148], MTA eribulin [190], and PBD [144,191,192,193,194]. Several of these ADCs are currently being investigated in clinical trials, and two ADCs with PEGylated linkers, loncastuximab tesirine and sacituzumab govitecan, have been FDA-approved for clinical use [143,195]. 

The advancement of self-immolative linker chemistry has also improved the stability and tolerability of ADCs utilizing cleavable linkers. In the early 1980s, the para-aminobenzyloxycarbonyl (PABC) self-immolate linker chemistry was developed by Carl et al. for use in prodrug design [196]. Since then, the PABC linker type has been extensively utilized in ADC linker technology, particularly for MMAE-conjugated ADC. Upon cleavage by lysosomal enzymes after ADC internalization into the target cells, the PABC spacer in the vc-PAB-MMAE linker/payload goes through a cascade of disassembly reactions and facilitates the release of untethered MMAE intracellularly [197]. The vc-PABC linker exhibits superior plasma stability and improved toxicity profile compared to the earlier cleavable linker generations, such as the pH-sensitive hydrazone linker used in calicheamicin-based ADCs or disulfide reducible linker used in DM1-based ADCs [198]. This linker technology has achieved significant success with its application in four MMAE-ADCs out of the twelve approved ADCs. In addition, the PABC spacer also has been used in the development of PBD-based ADCs, including the approved ADC loncastuximab tesirine. Recently, Pillow et al. have developed sophisticated self-immolative spacer chemistry that enabled direct conjugation of a cytotoxic payload to engineered cysteine residues [30]. This spacer facilitates a stable disulfide bridge, leading to enhanced plasma PK while allowing a rapid traceless release of free payloads intracellularly. This technology improved the tolerability of PBD-ADCs compared to the conventional peptide-PABC linker technology [199]. In this study, both the conventional peptide-ADC and the self-immolative disulfide-ADC displayed similar in vivo potency. However, while the self-immolative ADC was well-tolerated up to 10 mg/kg dose in rats, the animals treated with a 5 mg/kg dose of the peptide-ADC experienced significant weight loss.

Previous generations of cleavable peptide linkers, including the vc linker widely used in MMAE ADC technology, are susceptible to the peptidases present in plasma and in tissues. Recent developments in peptide-based linker technology have generated several linkers with appreciably enhanced stability against non-specific peptidase cleavage. For example, Chuprakov et al. introduced a β-glucuronide moiety to sterically protect the vc linker from non-specific peptidase cleavage in the systemic circulation [200]. Utilizing this technology, the authors developed an anti-CD79b ADC with a tandem-cleavage linker, which requires two sequential enzymatic cleavage events to release the cytotoxic payload: (1) removal of the monosaccharide group by β-glucuronidase present in the lysosomal compartment and, subsequently, (2) cleavage of the vc linkage by lysosomal peptidases. Compared to the anti-CD79b ADCs with a “standard” mono-cleavage vc-linker, the ADC with the tandem-cleavage linker technology demonstrated marked improvement in plasma stability and in vivo efficacy. In a tolerability study in rats, the animals treated with the mono-cleavage ADCs exhibited a significant reduction in circulating white blood cells, including monocytes, neutrophils, and eosinophils, whereas animals treated with the tandem-cleavage ADC exhibited minimal myelotoxicity. In another example, the Tsuchikama group developed tripeptide linkers with enhanced resistance to plasma and tissue-specific peptidases [201,202]. In a recent published study, they have optimized glutamic acid-glycine-citrulline (EGCit) linkers that have significantly improved resistance to neutrophil elastase compared to the conventional vc linker [202]. In an in vitro cytotoxicity study, treatment with an anti-HER2 EGCit-ADC had a minimal impact on cell viability of CD15+/CD66b+ differentiating human neutrophils, whereas treatment with an anti-HER2 ADC employing glutamic acid-valine-citrulline linker led to ~50% reduction in cell viability. In an in vivo toxicology study, mice treated with 80 mg/kg of the anti-HER2 EGCit-ADC exhibited minimal weight loss, whereas treatment with the same dose of an anti-HER2 ADC utilizing the conventional vc-linker led to ~15% weight loss. In addition, significant liver damage, as indicated by increased alanine aminotransferase (ALT), increased aspartate aminotransferase (AST), increased alkaline phosphatase (ALKP), and decreased blood urea nitrogen (BUN), was observed in the animals treated with 80 mg/kg of trastuzumab deruxtecan or trastuzumab emtansine, but not in animals treated with the same dose of the EGCit-ADC. 

The modification of the chemical structure of the payload may also lead to an improved ADC tolerability. For example, Miller et al. permutated the chemical structure of the DNA-crosslinking PBD-dimer to yield novel DNA-alkylating metabolites, indolinobenzodiazepine dimers (IGNs), through a reduction in one of the DNA-reactive imine functional groups [203]. IGN-conjugated ADCs exhibited an enhanced bystander effect (relative to PBD-conjugated ADCs), due to a more efficient release of free payload from the DNA adducts [204], which translated into better in vivo efficacy in murine tumor models [205]. In addition, mice tolerated IGN-conjugated ADCs at a 6 mg/kg dose compared to a 2.8 mg/kg dose for PBD-conjugated ADCs. At a 6 mg/kg dose, all of the mice treated with PBD-ADC (n = 8) experienced prolonged body weight loss and were dead by 60 days after treatment.

### 4.2. Antibody Modifications

Since most of the targets for ADCs also have some level of expression in healthy tissue [206], the delivery of the cytotoxic payload in these tissues through ADC binding can lead to on-target off-site toxicity. To reduce on-target toxicities, probody drug conjugates (PDC) have been developed where binding regions of the parent antibody are masked with anti-idiotypic peptides, which connect to the N-terminus of the light chain via a protease-susceptible peptide linker [207]. The intent is to enhance tumor selectivity through preferential activation within the tumor microenvironment, where upregulated protease activity [208,209] cleaves the masking peptide, thus enabling PDC binding to the target antigen. Slower cleavage of the masking peptide reduces the extent of PDC binding to the target antigen in healthy cells, and reduces on-target, off-site toxicity. In addition to potentially enabling selective activation within the tumor environment, PDC constructs, and complexes of ADC with anti-idiotypic binding domains, may enhance the ADC’s intra-tumoral distribution, efficacy, and therapeutic index by decreasing the impact of the “binding site barrier” within solid tumors [210,211,212,213]. In one notable preclinical example, Sagert et al. showed that a rodent Jagged antigen-binding PDC demonstrated substantially reduced weight loss relative to an ADC developed with the parent mAb, while the ADC and PDC produced similar efficacy [214]. Several PDC are under preclinical or clinical development [215,216,217,218]. 

Another approach to reducing on-target toxicity is through the development of bispecific antibodies targeting two tumor-associated antigens. This approach enables enhanced selectivity because avid ADC binding is only observed on cells with high co-expression of both antigens [219,220]. Indeed, work by Mazor et al. demonstrated a significant increase the in vitro binding selectivity of anti-HER2/EGFR bispecific antibodies to HER2+/EGFR+ cells than either HER2+/EGFR- or HER2-/EGFR+ cells [221]. A consistent finding was also observed with an anti-CD4/CD70 bispecific antibody. In addition, they also showed that binding selectivity could be further improved in bispecific antibodies with low-affinity variants of each binding arm, which exhibit poor binding to cells with single target expression [222,223]. Sellmann et al. demonstrated the feasibility of this dual-targeting concept in ADC technology with an MMAE-conjugated anti-EGFR/c-Met bispecific ADC [224]. EGFR is expressed widely, and skin toxicity is a significant concern with EGFR targeting therapies [225,226]. Compared to the anti-EGFR MMAE-conjugated cetuximab, the bispecific ADC displayed reduced in vitro binding activity and reduced cellular cytotoxicity when applied to healthy cells (e.g., HepG2 and human keratinocytes), while demonstrating high potency in tumor cells that express both targets (EGFR and c-Met) [224]. Several additional bispecific ADCs have been recently reported, including anti-HER2/integrin MMAF-conjugated bispecific ADCs [227], an anti-HER2/CD63 bispecific ADC [228], and an anti-HER2/PRLR bispecific ADC [229].

### 4.3. Modifying Dosage Regimens

Modifying clinical dosing schedules through fractional dosing has proven to improve the therapeutic index of ADCs in clinical settings. For instance, gemtuzumab ozogamicin was initially approved in 2001 to treat CD33-positive AML at the dose of 9 mg/m^2^ given at least two weeks apart. However, a high incidence of severe clinical toxicities was observed, including thrombocytopenia (99%), neutropenia (97%), grade ≥ 3 hyperbilirubinemia (23%), and grade ≥ 3 elevated alanine transaminase or aspartate transaminase levels, while no significant improvements in efficacy were shown compared to the standard of care [80]. Consequently, gemtuzumab ozogamicin was withdrawn from the market in 2010. Recently, fractionated dosing of the ADC was introduced such that patients receive the dose of 9 mg/m^2^ over three doses of 3 mg/m^2^ on the 1st, then 4th, and the 7th days of the dosing cycle, resulting in a more favorable toxicity profile [84]. PK/PD analysis of gemtuzumab ozogamicin indicates that its toxicities are driven by peak plasma concentrations, while its efficacy is driven by exposure [8,230]. Therefore, fractionated dosing would decrease the peak plasma concentrations and reduce toxicities while the maintaining drug exposure levels. With the modified dosing regimen, the ADC was re-approved for clinical use in 2017.

### 4.4. Inverse Targeting Strategy

The Balthasar laboratory has recently introduced the use of payload binding antibodies for “inverse targeting” to improve the therapeutic selectivity of ADCs. Briefly, payload binding antibody fragments (a.k.a. payload binding selectivity enhancers or PBSE) are co-administered with ADCs to enable the binding, neutralization, and clearance of released payload from plasma, decreasing the exposure of released payload in tissues associated with ADC toxicities. Antibody fragments have been utilized extensively to mitigate the adverse effects of drugs and toxins [231,232], including the use of anti-drug antibodies to enhance the pharmacokinetic selectivity of regional chemotherapy [233,234,235]. For example, the subcutaneous administration of anti-methotrexate Fab fragments significantly increased the maximum tolerated dose of intraperitoneal methotrexate by more than 5-fold (from 1.9 mg/kg to 10 mg/kg) in mice bearing peritoneal tumors [236]. Additionally, intravenous anti-topotecan antibody therapy allowed a significant increase in the MTD of intraperitoneal topotecan in a mouse model of peritoneal cancer [237]. The application of PBSE for the inverse targeting of ADCs has been recently pursued by the Balthasar group as a means of enhancing the safety and therapeutic index of ADCs. ABC3315, an MMAE payload binding selectivity enhancer (PBSE) Fab fragment, selectively binds to free MMAE but not to ADC-conjugated MMAE [238]. In in vitro cell culture, the co-incubation of ABC3315 with polatuzumab vedotin or trastuzumab-vc-MMAE led to an increase in the selectivity ratio of the ADC potency/payload potency by 738-fold in Ramos lymphoma cells and by 385-fold in SKBR3 breast cancer cells. In an in vivo lymphoma animal model, the co-treatment of polatuzumab vedotin with ABC3315 did not reduce the anti-tumor efficacy of the ADC; however, the co-dosing of ABC3315 with 120 mg/kg polatuzumab vedotin at a 3-fold molar ratio relative to MMAE significantly decreased the nadir body weight loss from 11.86% (for ADC plus PBS treated group) to 4.13% (for ADC plus ABC3315 treated group) in Swiss-Webster mice. Similarly, an anti-DM4 PBSE single-domain antibody, DMOH9, was developed for the inverse targeting of DM4-conjugated ADCs [239]. Co-dosing DMOH9 with 55 mg/kg 7E7-DM4, an anti-CD123-SPDB-DM4 ADC, at a 10-fold molar ratio relative to DM4 significantly decreased the nadir body weight loss from 11.86% (for ADC plus PBS treated group) to 4.13% (for ADC plus DMOH9 treated group) in Swiss-Webster mice. In an AML mouse model, the co-treatment of 7E7-DM4 with DMOH9 did not reduce the efficacy of the ADC or the survival of the tumor-bearing mice compared to the treatment with 7E7-DM4 alone at 1 or 10 mg/kg. Administration of the ADC (alone) at 100 mg/kg led to rapid death in 80% of treated mice; however, co-administration of the PBSE enabled all mice to tolerate this dose of the ADC, and allowed dramatically enhanced anti-tumor activity and animal survival. The PBSE technology may have utility in mitigating many off-site toxicities mediated by released payload, potentially leading to substantially improved ADC safety and efficacy.

## 5. Conclusions

By targeting the differential expression of cell-surface antigens on cancer cells vs. healthy cells, antibody-drug conjugates aim to increase the selectivity of the delivery of anti-cancer agents, and to widen the therapeutic window of traditional chemotherapy. However, the clinical utility of ADCs is limited by dose-limiting off-site toxicities. Significant research efforts have been invested to understand ADC-mediated off-site toxicity, and to develop strategies to improve ADC tolerability and, thus, efficacy.

## Figures and Tables

**Table 1:** Summary of the approved ADCs and their common adverse events.

Approved ADCs	Target	Indications	Approved Year/ R&D Organization	Common Adverse Events (Any Grades)	Common Grade ≥ 3 Adverse Events
Gemtuzumab Ozogamicin (Mylotarg^®^)	CD-33	Acute myeloid leukemia	2001 Pfizer	Thrombocytopenia, fatigue, neutropenia, pyrexia, nausea, infection, chills, hemorrhage, vomiting, headache, stomatitis, diarrhea, and abdominal pain	Neutropenia, thrombocytopenia, increased AST/ALT levels, and sepsis
Inotuzumab Ozogamicin (Besponsa^®^)	CD-22	B-cell precursor acute lymphoblastic leukemia	2017 Pfizer	Neutropenia, thrombocytopenia, infection, anemia, leukopenia, febrile neutropenia, and nausea	Neutropenia, thrombocytopenia, leukopenia, febrile neutropenia, anemia, and lymphopenia
Brentuximab Vedotin (Adcetris^®^)	CD-30	Hodgkin lymphoma, systemic anaplastic large-cell lymphoma, T-cell lymphoma	2011 Seattle Genetics	Peripheral sensory neuropathy, nausea, fatigue, neutropenia, diarrhea, pyrexia, vomiting, arthralgia, pruritus, myalgia, peripheral motor neuropathy, and alopecia	Neutropenia, peripheral sensory neuropathy, thrombocytopenia, and anemia
Polatuzumab Vedotin (Polivy^®^)	CD-79b	Diffuse large B-cell lymphoma	2019 Genentech	Neutropenia, anemia, and peripheral neuropathy	Neutropenia, anemia, and peripheral sensory neuropathy
Enfortumab Vedotin (Padcev^®^)	Nectin-4	Urothelial cancer	2019 Astellas	Fatigue, alopecia, decreased appetite, dysgeusia, nausea, peripheral sensory neuropathy, pruritus, diarrhea, and maculopapular rash	Rash, neutropenia, anemia, and fatigue
Tisotumab Vedotin (Tivdak^®^)	Tissue factor	Cervical cancer	2021 Genmab	Epistaxis, fatigue, nausea, alopecia, conjunctivitis, decreased appetite, constipation, diarrhea, vomiting, peripheral neuropathy, dry eye, and abdominal pain	Fatigue, anemia, abdominal pain, hypokalemia, conjunctivitis, hyponatremia, peripheral neuropathy, and vomiting
Belantamab Mafodotin (Blenrep^®^)	B-cell maturation antigen	Multiple myeloma	2020 GSK	Keratopathy, thrombocytopenia, anemia, nausea, pyrexia, blurred vision, increased aspartate aminotransferase	Keratopathy, thrombocytopenia, anemia
Trastuzumab Emtansine (Kadcyla^®^)	HER-2	Breast cancer	2013 Genentech	Thrombocytopenia, elevated transaminases, fatigue, anemia, and nausea	Thrombocytopenia, increased aspartate aminotransferase levels, and anemia
Mirvetuximab Soravtansine (Elahere^®^)	Folate receptor α	Ovarian cancer	2022 Immunogen	Nausea, blurred vision, keratopathy, diarrhea, fatigue, peripheral neuropathy, dry eye, and decreased visual acuity	Blurred vision, peripheral neuropathy, and diarrhea
Trastuzumab Deruxtecan (Enhertu^®^)	HER-2	Breast cancer	2019 Daiichi Sankyo	Nausea, fatigue, alopecia, vomiting, neutropenia, constipation, anemia, decreased appetite, diarrhea, leukopenia, and thrombocytopenia	Neutropenia, anemia, nausea, leukopenia, lymphopenia, and fatigue
Sacituzumab Govitecan (Trodelvy^®^)	Trop-2	Breast cancer, urothelial cancer	2020 Gilead Sciences	Nausea, diarrhea, neutropenia, fatigue, vomiting, anemia, alopecia, and constipation	Neutropenia, anemia, diarrhea, and leukopenia
Loncastuximab Tesirine (Zynlonta^®^)	CD-19	Large B-cell lymphoma	2021 ADC Therapeutics	Neutropenia, thrombocytopenia, anemia, fatigue, and gamma-glutamyl transferase increase	Thrombocytopenia, neutropenia, anemia, gamma-glutamyl transferase increased, leukopenia, lymphopenia, and hypophosphatemia

**Table 2:** Frequently reported dose-limiting toxicities associated with payload classes.

	Approved/Late-Stage ADCs	Linker Type	Key Toxicities
Tubulin inhibitors			
MMAE	Brentuximab Vedotin, Polatuzumab Vedotin, Enfortumab Vedotin, Tisotumab Vedotin, Disitamab Vedotin	Cleavable	Neutropenia, peripheral neuropathy, anemia, skin toxicity
MMAF	Belantamab Mafodotin	Non-cleavable	Thrombocytopenia, ocular toxicity, hepatic toxicity
DM1	Trastuzumab Emtansine	Non-cleavable	Thrombocytopenia, hepatic toxicity
DM4	Mirvetuximab Soravtansine	Cleavable	Neutropenia, anemia, peripheral neuropathy, ocular toxicity
DNA-crosslinkers/ DNA-alkylators			
Calicheamicin	Gemtuzumab Ozogamicin, Inotuzumab Ozogamicin	Cleavable	Neutropenia, thrombocytopenia, hepatic toxicity
PBD	Loncastuximab Tesirine	Cleavable	Neutropenia, thrombocytopenia, anemia, serosal effusion, nephron toxicity, skin toxicity
Duocarmycin	Trastuzumab Duocarmazine	Cleavable	Neutropenia, thrombocytopenia, serosal effusion
Topoisomerase inhibitors			
SN-38	Sacituzumab Govitecan	Cleavable	Neutropenia, gastrointestinal toxicity
Deruxtecan	Trastuzumab Deruxtecan	Cleavable	Neutropenia, gastrointestinal toxicity
